# Surgical Management of Infective Endocarditis: Indications, Techniques, and Outcomes

**DOI:** 10.7759/cureus.91381

**Published:** 2025-09-01

**Authors:** Abubakar I. Sidik, Maxim L Khavandeev, Vladislav V Dontsov, Grigorii A Esion, Md Limon Hossain, Emmanuel Joachim Njoya Mbombo, Ivan Karpenko, Derrar Ahlam

**Affiliations:** 1 Cardiovascular Surgery, Peoples' Friendship University of Russia, Moscow, RUS; 2 Cardiovascular Surgery, Gusak Institute of Emergency and Reconstructive Surgery, Donetsk, RUS; 3 Cardiovascular Surgery, Moscow Regional Research and Clinical Institute, Moscow, RUS; 4 Cardiovascular Surgery, A.A. Vishnevskiy Hospital, Moscow, RUS; 5 Cardiology, I.M. Sechenov First Moscow State Medical University, Moscow, RUS; 6 Cardiovascular Medicine, Peoples' Friendship University of Russia, Moscow, RUS

**Keywords:** heart valve replacement, infective endocarditis, periannular abscess, postoperative management, prosthetic valve infective endocarditis, surgical indications, valve repair

## Abstract

Infective endocarditis (IE) remains a complex condition with high morbidity and mortality despite advances in antimicrobial therapy and diagnostic imaging. Surgery is required in up to half of patients, most commonly for heart failure, uncontrolled infection, or prevention of embolic events. Surgical timing, ranging from emergency to elective, is influenced by hemodynamic compromise, neurological status, pathogen virulence, and vegetation characteristics. Modern imaging modalities, including transesophageal echocardiography, cardiac CT, and 18F fluorodeoxyglucose positron emission tomography/computed tomography (18F-FDG-PET/CT), play a central role in surgical decision-making by detecting complications and guiding operative strategies. Intraoperative management focuses on radical debridement and restoration of valve function, with repair, replacement, or complex reconstructions tailored to the extent of infection and patient-specific factors. Prosthesis selection remains individualized, as no single valve substitute demonstrates clear superiority in survival, reinfection, or reoperation rates. Postoperative management requires prolonged antimicrobial therapy, vigilant follow-up, and rehabilitation, while recurrence risk highlights the importance of multidisciplinary care. Long-term prognosis depends on underlying comorbidities, pathogen type, and patient adherence to preventive measures. Future research should address gaps in surgical timing after neurological events, management of prosthetic and transcatheter-associated IE, and strategies for improving outcomes in high-risk populations.

## Introduction and background

Infective endocarditis (IE) remains one of the most complex and high-risk conditions in cardiovascular medicine, characterized by infection of the endocardial surfaces, most often the heart valves. Despite advances in antimicrobial therapy, diagnostic imaging, and supportive care, IE continues to carry substantial morbidity and mortality, particularly when complicated by heart failure, uncontrolled infection, or systemic embolism [1]. The global burden of the disease has evolved, with rising incidence among patients with prosthetic valves, cardiac implantable electronic devices (CIEDs), and congenital heart disease, as well as in vulnerable populations such as people who inject drugs (PWID) and the immunocompromised [2,3]. The epidemiology of IE continues to evolve, with an annual incidence of three to 10 cases per 100,000 individuals globally, driven by shifts from rheumatic heart disease in low-resource settings to degenerative valve issues, device implantation, and an aging population (average age greater than 70 years) in developed nations. In high-income countries, IE is increasingly associated with healthcare-related factors, such as prosthetic valves, CIED, and degenerative valve disease in aging populations, with rising cases due to *Enterococcus faecalis* and coagulase-negative staphylococci (CoNS) in the elderly [3]. In contrast, low- and middle-income countries face barriers, such as socioeconomic challenges and limited health-system access, where over 40 million individuals with rheumatic heart disease, a key risk factor, reside. In these regions, children account for over 30% of cases.

Prosthetic valve IE now comprises 20% to 30% of cases, contributing to higher mortality rates (18% in-hospital, 30% at six months), particularly with *Staphylococcus aureus* infections and comorbidities [4]. While antimicrobial therapy is the cornerstone of medical management, surgery plays an essential role in the treatment of IE, especially in cases with structural damage, persistent sepsis, or risk of embolization. In appropriately selected patients, surgical intervention can be life-saving by relieving hemodynamic compromise, eradicating infection, and preventing devastating complications [5,6]. However, the decision to operate is often nuanced, requiring careful integration of clinical, microbiologic, and imaging data. Additionally, the surgical landscape in IE is fraught with technical challenges, including tissue destruction, prosthesis choice, and timing, particularly in the context of neurologic complications or multiple comorbidities [6,7].

This review explores the role of surgery in the management of IE across the continuum of care. It outlines the key indications for surgical intervention, details intraoperative strategies and decision-making, and examines long-term outcomes after discharge. The goal is to provide a comprehensive and up-to-date synthesis that supports clinicians in navigating the complexity of surgical management in IE and highlights the importance of multidisciplinary collaboration for optimal outcomes.

## Review

Methodology

A comprehensive literature search was conducted to identify relevant studies on the surgical management of IE. The search was performed in PubMed, Scopus, and Web of Science databases for the period from January 2015 to January 2025. The following Medical Subject Headings (MeSH) and keywords were used in various combinations: “endocarditis, bacterial”, “heart valve diseases/surgery”, “cardiac surgical procedures”, “heart valve prosthesis implantation”, “heart valve repair”, “valve replacement, heart”, “prosthesis-related infections”, “abscess/surgery”, “reoperation”, “treatment outcome”, “postoperative complications”, and “heart failure/surgery”. Boolean operators (AND, OR) were applied to combine terms, and additional keywords such as “infective endocarditis”, “surgical management”, “valve repair”, and “valve replacement” were included to ensure a comprehensive search.

The initial search yielded 275 articles. After limiting to original articles and guidelines published in English, the number was reduced to 155. Studies were further screened for relevance to the surgical management of IE, resulting in 46 articles being included for detailed analysis. Conference abstracts, case reports, editorials, reviews, and expert opinions were excluded.

For the included studies, data were extracted from the main text, tables, and figures. Additionally, the references cited within these articles were reviewed, and any information deemed relevant to the topic was incorporated. The extracted data were used to synthesize current evidence regarding surgical indications, techniques, outcomes, and postoperative considerations in IE.

Indications for surgery in infective endocarditis

The decision to pursue surgical intervention in IE hinges on a balance between the risks of operative mortality and the potential to prevent irreversible complications or death from the infection itself. Surgery is indicated in up to 50% of IE cases, most often due to progressive heart failure, uncontrolled infection, or the threat of embolic events. The appropriate identification and timing of these indications are central to optimizing patient outcomes [8].

Recent guidelines underscore multidisciplinary endocarditis teams for optimizing surgical timing and managing complications. As summarized in Table 1, European Society of Cardiology (ESC) and American Association for Thoracic Surgery (AATS) recommendations align on emergency/urgent surgery for heart failure (e.g., cardiogenic shock from regurgitation/fistula) and uncontrolled infection (e.g., persistent bacteremia or fungal/resistant organisms), with urgent intervention for large vegetations (>10 mm) post-embolism or right-sided IE with severe malfunction or emboli [2,9].

**Table 1:** Surgical Indications for Infective Endocarditis According to European Society of Cardiology (ESC) 2015 and American Association for Thoracic Surgery (AATS) 2016 Guidelines Classes of Recommendation — I: strong recommendation; IIa: moderate recommendation; IIb: weak recommendation. Levels of Evidence — A: high-quality evidence from multiple randomized trials or meta-analyses; B: moderate-quality evidence from a single randomized trial or large non-randomized studies; C: consensus of experts or small/retrospective studies.

Indication	ESC Guidelines 2015	AATS Guidelines 2016
Heart Failure	In cases of aortic or mitral native valve endocarditis (NVE) or prosthetic valve endocarditis (PVE) with severe acute regurgitation, obstruction, or fistula resulting in cardiogenic shock (EMERGENCY, I, B) or with compromised hemodynamic stability (URGENT, I, B).	Recommended when IE is associated with valve dysfunction producing symptoms of heart failure (I, B).
Uncontrolled Infection	Persistent local infection requiring immediate surgical management (URGENT, I, B); infections caused by fungi or highly resistant pathogens (URGENT/ELECTIVE, I, C); ongoing positive blood cultures despite adequate antibiotic therapy and proper treatment of secondary septic sites (URGENT, IIa, B); PVE due to staphylococcal or non-HACEK Gram-negative organisms (URGENT/ELECTIVE, IIa, C).	Surgery advised for patients with left-sided IE caused by *Staphylococcus aureus*, fungal organisms, or other highly resistant microbes (I, B). Also indicated for destructive penetrating lesions (I, B) or persistent bacteremia/fever lasting beyond five to seven days despite appropriate antimicrobial treatment (I, B). Includes management of PVE and recurrent infections (IIa, C).
Prevention of Embolism	For NVE or PVE with vegetations >10 mm after one or more embolic episodes despite proper antibiotics (URGENT, I, B). Also indicated in severe valve stenosis/regurgitation with embolic potential (URGENT, I, B). Isolated very large vegetations (>30 mm) (URGENT, IIa, B). Cases of aortic/mitral NVE or PVE with vegetations >15 mm and no other surgical indications (URGENT, IIb, C).	Consider surgery for recurrent embolic events and persistent vegetations despite antibiotics (IIa, B). NVE or PVE with mobile vegetations >10 mm accompanied by embolic events after suitable antimicrobial therapy should receive immediate or urgent surgery (IIb, B).
Right-sided Infective Endocarditis	Microbes that are difficult to eradicate or bacteremia persisting >7 days despite antibiotic therapy; tricuspid vegetations >20 mm with repeated pulmonary emboli, regardless of the presence of right heart failure (IIa, C).	NVE or PVE with symptomatic valve dysfunction, large vegetations, or persistent infection presenting with continuous bacteremia or fever for five to seven days despite therapy; also indicated for septic pulmonary embolism (IIb, B).

Role of emerging diagnostic modalities in surgical decision-making

Transthoracic echocardiography (TTE) and transesophageal echocardiography (TOE) remain first-line for detecting valvular lesions, vegetations, and complications like perivalvular abscesses or fistulae, with TOE recommended in suspected cases even if TTE is negative. Advanced echocardiography variants, such as intracardiac echocardiography, are useful in challenging scenarios (e.g., prosthetic valve endocarditis (PVE), transcatheter aortic valve implantation (TAVI)/transcatheter pulmonary valve implantation (TPVI) IE, or CIED-related IE) to confirm vegetations or lead infections when TTE/TOE is nondiagnostic [9].

Emerging modalities like 18F-fluorodeoxyglucose positron emission tomography/computed tomography (18F-FDG-PET/CT) and white blood cell single photon emission tomography/computed tomography (WBC SPECT/CT) add value by detecting metabolic activity indicative of infection, especially in PVE (sensitivity 86%, specificity 84%) or CIED IE, where echocardiography may miss up to 30% of cases. 18F-FDG-PET/CT is recommended in possible PVE to confirm diagnosis and identify extracardiac foci (e.g., spondylodiscitis, septic emboli), potentially reclassifying cases from "possible" to "definite" IE per modified ESC criteria. Cardiac CT angiography complements by visualizing paravalvular extensions, pseudoaneurysms, or abscesses, influencing surgical planning in 33% of TAVI IE cases. Whole-body CT or PET/CT aids in detecting silent emboli (e.g., splenic, cerebral), which occur in 20-50% of patients and guide the urgency of intervention [9].

In surgical decision-making, these modalities help assess infection extent and complications (e.g., uncontrolled infection, heart failure, embolic risk), key indications for surgery (performed in up to 50% of cases). For instance, PET/CT identifies periprosthetic destruction in PVE, prompting complex reconstructions like root replacement, while detecting extracardiac sites (e.g., osteoarticular infections in 6-8% of cases) ensures pre-surgical management to reduce recurrence. Intra-operative TOE provides real-time guidance on debridement and prosthesis choice [9].

Timing and classification of surgery

Surgical timing in IE is stratified into three broad categories-emergency, urgent, and elective-based on the clinical urgency and severity of the disease (Table 2). Determining the appropriate timing requires an assessment of hemodynamic stability, extent of infection, and risk of embolic complications [10].

**Table 2:** Categories of Surgical Timing in Infective Endocarditis

Timing Category	Definition	Common Indications
Emergency	Surgery required within hours	Cardiogenic shock; severe acute valvular insufficiency; rapid hemodynamic deterioration
Urgent	Surgery required within days	Uncontrolled infection; high embolic risk; moderate heart failure
Elective	Surgery can be scheduled after medical stabilization	Persistent vegetations; indications not immediately life-threatening

Factors determining surgical urgency include the severity of valvular destruction, degree of heart failure, risk of systemic embolization, presence of intracardiac abscesses, and the virulence of the infecting organism. Multidisciplinary input is critical in prioritizing surgical timing, especially in patients with comorbidities or neurological complications [11].

Significant gaps exist in the evidence base concerning the optimal timing of surgery in patients with IE complicated by neurological events, primarily due to the lack of randomized controlled trials (RCTs), with most insights relying on observational studies or expert consensus (Level C evidence). The timing of surgery after an ischemic stroke or intracranial hemorrhage presents a challenging risk-benefit balance between preventing further embolic events and avoiding exacerbation of neurological damage. Current suggestions recommend delaying surgery for at least four weeks after a major ischemic stroke or any intracranial hemorrhage to reduce the risk of worsening cerebral injury, though this is supported by limited data and may not apply uniformly across all cases [9,10].

For instance, urgent surgery may be considered within 72 hours in patients with life-threatening heart failure or uncontrolled infection despite a recent stroke, yet there is insufficient evidence to define precise thresholds for risk stratification or to standardize protocols. Additionally, the impact of cerebral microbleeds or silent emboli detected by advanced imaging on surgical timing remains underexplored. Further research, including RCTs and prospective registries, is needed to establish evidence-based timing algorithms and address these critical uncertainties, particularly in balancing hemodynamic stability with neurological outcomes [9,10].

Heart Failure and Hemodynamic Compromise

Heart failure is the most frequent indication for surgery in IE and often represents the turning point in clinical decision-making. Acute regurgitation due to leaflet perforation, chordal rupture, or prosthetic dehiscence can result in pulmonary edema and cardiogenic shock [12,13]. The aortic and mitral valves are most commonly involved, and their dysfunction can precipitate rapid hemodynamic deterioration. The clinical presentation may range from new-onset heart failure to refractory symptoms unresponsive to medical therapy [14].

**Figure 1:**
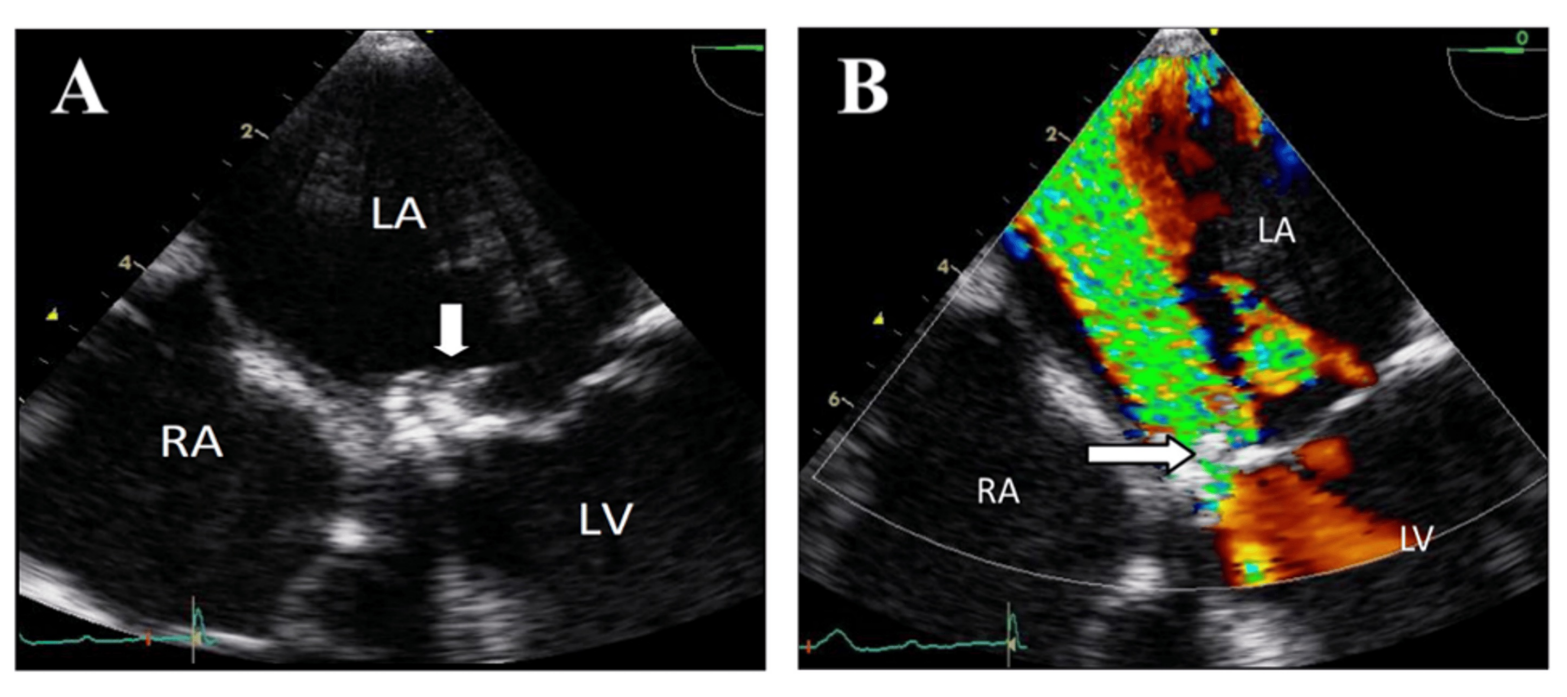
Transesophageal Echocardiograms Illustrating Mitral Valve Perforation in Infective Endocarditis A. Mid-esophageal four-chamber view showing perforation of the anterior mitral valve leaflet with a “windsock” appearance (arrow). B. Same view with color Doppler interrogation, demonstrating severe mitral regurgitation through the perforation (arrow). LA - left atrium; LV - left ventricle; RA - right atrium Reprinted under the terms of the Creative Commons Attribution 4.0 International License from Bonou et al. [15].

Surgical repair or replacement is warranted when the degree of valve dysfunction leads to severe heart failure, particularly when accompanied by echocardiographic evidence of flail leaflet, large vegetations, annular disruption, or leaflet perforation. A striking example is the “windsock” appearance of an anterior mitral valve leaflet perforation caused by *Staphylococcus aureus*, leading to severe regurgitation and acute pulmonary edema (Figure 1) [15]. Early surgery in this setting improves survival and should not be delayed for completion of antibiotic therapy [16,17].

Uncontrolled or Persistent Infection

**Figure 2:**
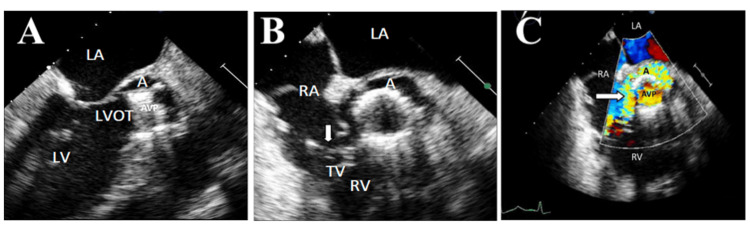
Transesophageal Echocardiograms Illustrating Perivalvular Complications in Infective Endocarditis A. Three-chamber view showing a ruptured abscess forming a pseudoaneurysm adjacent to the aortic valve prosthesis (AVP). B. Mid-esophageal short-axis view at the aortic valve level, showing a large pseudoaneurysm around the AVP annulus and vegetations on the tricuspid valve (TV). C. Same view as B with color Doppler, demonstrating flow into the pseudoaneurysm and a fistula between the pseudoaneurysm and the RA. LA - left atrium; LV - left ventricle; LVOT - left ventricular outflow tract; RA - right atrium; RV - right ventricle Reprinted under the terms of the Creative Commons Attribution 4.0 International License from Bonou et al. [15].

Failure to sterilize the infection despite appropriate antibiotic therapy is a hallmark of uncontrolled IE and necessitates surgical intervention. Indicators include persistent bacteremia, rising inflammatory markers, and systemic signs of sepsis [18,19]. Intracardiac complications such as annular abscesses, pseudoaneurysms, and fistulous tracts are strong predictors of surgical need. These lesions often escape medical management and pose a high risk of extension, rupture, or recurrence [8,17]. Such complications significantly increase mortality in IE and often require complex surgical intervention, such as the commando procedure for aorto-mitral continuity (AMC) reconstruction. TOE is critical for identifying these lesions, as illustrated in a case of PVE with a mitral-aortic intervalvular fibrosa pseudoaneurysm and left ventricular outflow tract (LVOT)-to-right atrium fistula (Figure 2) [15].

Surgery is also indicated for infections caused by highly virulent or resistant organisms, such as methicillin-resistant *Staphylococcus aureus* (MRSA), *Candida* spp., and multidrug-resistant gram-negative bacteria, which are associated with poor response to antibiotics and high relapse rates [17,19].

Prevention of Systemic Embolism

Embolic events are a significant complication in IE, occurring in 20% to 50% of cases and often affecting the brain, spleen, or lungs. These events are predominantly caused by the migration of cardiac vegetations, with the highest incidence observed within the first two weeks of initiating antibiotic therapy. Assessing embolic risk relies on evaluating vegetation characteristics, such as size, mobility, and location, alongside pathogen type and other clinical factors to guide preventive strategies, including potential surgical intervention. Vegetation size, measured as the maximal length via echocardiography, is a key predictor and helps inform decisions about when surgery might be necessary [20,21].

Key thresholds for vegetation size include greater than or equal to 10 mm, which is widely recognized as a marker of elevated embolic risk, especially for new or recurrent events. Persistent vegetations of this size after one or more embolic episodes, despite appropriate antibiotic treatment, typically justify urgent surgery within three to five days in cases of aortic or mitral native valve endocarditis (NVE) or PVE. This threshold also supports urgent intervention when combined with other surgical indications like heart failure or uncontrolled infection. For isolated vegetations greater than or equal to 10 mm without severe valve dysfunction, clinical embolism, or high surgical risk, urgent surgery may be considered in low-risk patients, though this recommendation is less definitive. Vegetations exceeding 30 mm are associated with particularly high rates of neurological complications, necessitating prompt evaluation. Additionally, progressive enlargement during therapy signals uncontrolled infection and increased embolic potential, further supporting the case for urgent surgery. In right-sided IE, a vegetation size less than or equal to 20 mm, combined with a good response to antibiotics, no metastatic sites, and no complications, may allow for shorter two-week antibiotic regimens or conservative management due to lower risk. Other factors influencing risk include vegetation mobility, which amplifies danger regardless of size and location, with mitral valve involvement, especially on the anterior leaflet, posing a greater embolic threat than aortic involvement. The type of pathogen, such as *Staphylococcus aureus*, *Streptococcus gallolyticus*, or *Candida* species, also heightens risk, often overshadowing size alone. Additional considerations include prior embolism, multivalvular disease, atrial fibrillation, diabetes, and elevated biological markers like D-dimer, which further elevate the embolic risk profile [22,23].

Relying solely on vegetation size for surgical decisions may be insufficient unless paired with other critical conditions, as outcomes may not improve otherwise. The evidence base indicates that embolic rates decrease after antibiotic initiation, and pathogen-specific risks, such as those from S. aureus, remain critical. Future research should focus on refining these cut-offs through prospective trials, incorporating multimodality imaging for more precise risk stratification [24,25].

Surgical techniques and intraoperative considerations

Preoperative Evaluation

Thorough preoperative assessment is fundamental in the surgical management of IE [26,27]. Imaging plays a pivotal role in defining the extent of valvular destruction, identifying perivalvular complications, and guiding surgical planning. TOE remains the primary diagnostic modality, providing detailed visualization of vegetations, leaflet perforations, abscesses, and fistulae. In cases where TOE findings are inconclusive, particularly in prosthetic valve or device-related endocarditis, complementary imaging with computed tomography or nuclear modalities such as FDG-PET/CT or WBC-SPECT/CT can help detect paravalvular extension and extracardiac septic foci [27].

Coronary artery assessment is also a critical preoperative step in patients with risk factors for atherosclerosis or those over 40 years of age [28]. When feasible, this can be achieved via CT coronary angiography or conventional invasive angiography, depending on the clinical context and urgency [29]. Additionally, preoperative evaluation must include a search for extracardiac sources of infection, such as spinal osteomyelitis, splenic abscesses, or indwelling catheter-related infections. Identifying and treating these foci is essential to reducing the risk of persistent or recurrent infection after surgery.

Intraoperative Strategies

Intraoperative management of IE aims to completely remove infected tissue and restore normal cardiac anatomy and hemodynamic function. The decision between valve repair and valve replacement is guided by the extent of structural destruction, the acuity of the infection, and patient-specific factors such as age, comorbidities, and anticoagulation tolerance. In all cases, excised tissue should be sent for pathological, microbiological, and molecular analyses to guide ongoing antimicrobial therapy [30,31].

For aortic valve involvement, replacement is generally required, as repair is rarely feasible in the acute setting. In select cases, such as isolated aortic regurgitation after healed infection, repair may be considered, although these are uncommon [32,33]. Mitral valve disease caused by endocarditis can sometimes be addressed with repair techniques, particularly when leaflet perforations occur with intact free margins and chordae tendineae. These situations often allow patch repair, especially in subacute or healed infections [31,34]. More complex mitral valve involvement, affecting the annulus, leaflet free edge, or chordae, may also be amenable to repair in expert hands, but evidence on long-term durability remains limited. Comparative data between repair and replacement are difficult to interpret due to differences in patient characteristics and infection severity, so no definitive superiority can be concluded. In the acute setting, repair should only be performed if a durable outcome is expected and complete eradication of infection is achievable [30,31,35]. In children, repair may be prioritized because replacement options are more limited [10].

**Figure 3:**
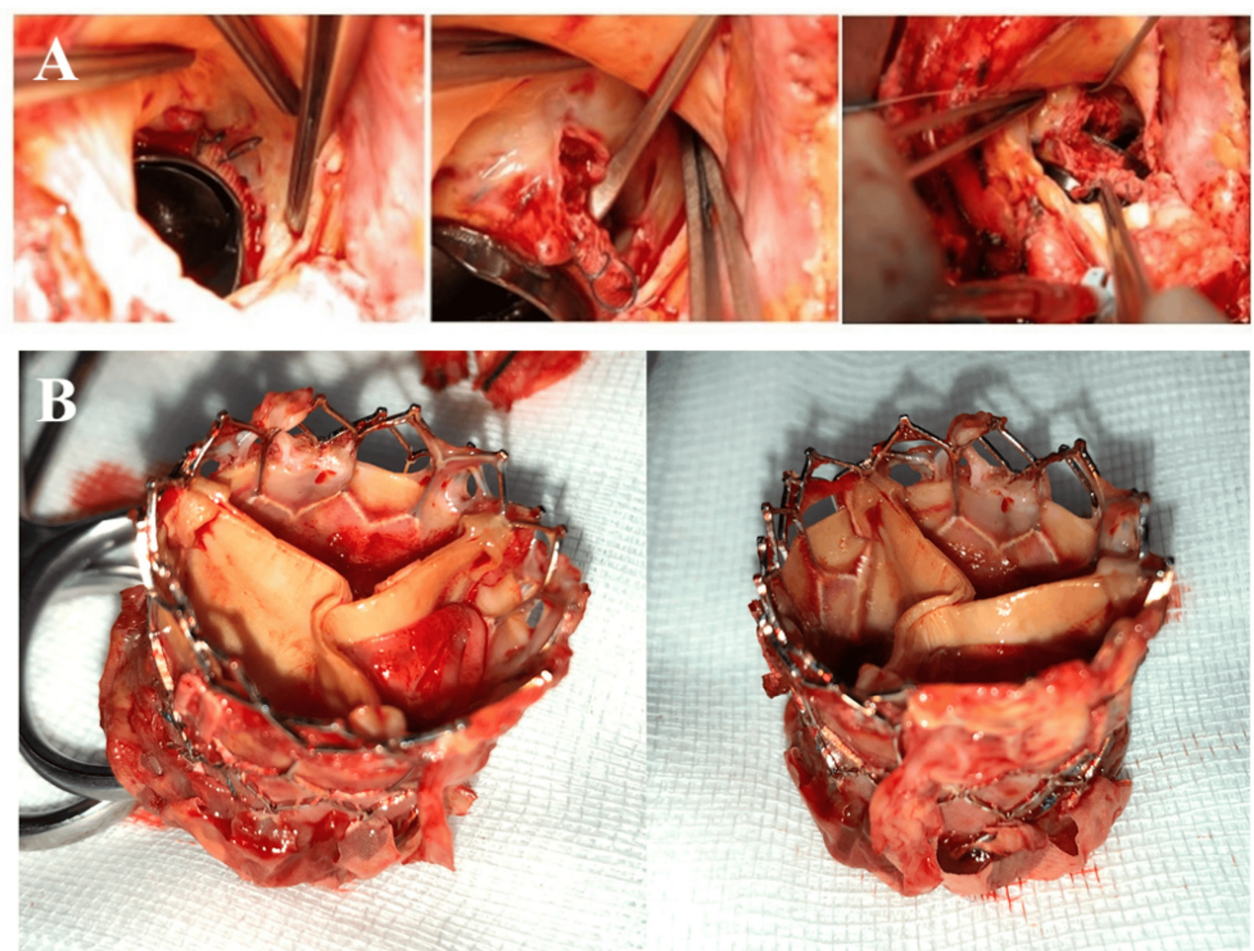
Prosthetic Aortic Valve Endocarditis A. Intraoperative picture of mechanical prosthetic aortic valve endocarditis and aortic annular erosion and abscess, detected during the prosthesis removal. B. Intraoperative image of explanted transcatheter aortic valve implantation (TAVI) prosthesis in a patient presenting with acute infective endocarditis following TAVI. Reprinted under the terms of the Creative Commons Attribution 4.0 International License from Iaccarino et al. [36] and Weymann et al. [37], respectively.

When the infection involves the aortic annulus, the surgical approach depends on the extent of tissue damage. Limited periannular involvement, such as shallow abscesses or small pseudoaneurysms, can often be managed with standard valve replacement [33]. Aortic annular erosion and abscess can be detected in both native and prosthetic aortic valve endocarditis (Figure 3) [36,37]. More extensive disease with aortic root abscess or major periannular destruction usually requires aortic root replacement. In experienced centers, allografts are often chosen for their ability to adapt to irregular tissue surfaces, provide excellent hemodynamics, and lower thromboembolic risk, while also enabling reconstruction of adjacent structures such as the anterior mitral leaflet [32,38]. Stentless bioprostheses offer similar advantages, especially in patients with small aortic roots, and have been associated with low reinfection rates, though strong comparative evidence is lacking [32]. In selected cases, particularly in pediatric patients, the Ross procedure (pulmonary autograft) may be an appropriate alternative [10].

**Figure 4:**
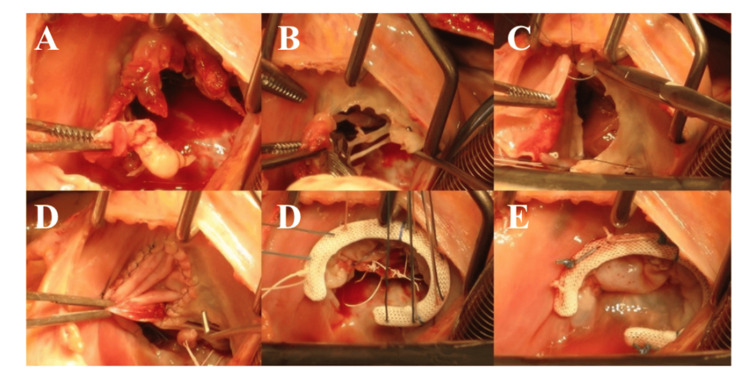
Intraoperative Picture of Native Tricuspid Valve Infective Endocarditis Through right atriotomy, exposure of tricuspid valve. A, B. Large vegetation is detected and resected on the anterior leaflet. C, D. Reconstruction of the leaflet is obtained through a pericardial patch. E. Neochordal apparatus is sutured on the free margin of the new anterior leaflet, and in order to optimize coaptation and competence, an incomplete tricuspid annular ring is positioned. F. Final result. Reprinted under the terms of the Creative Commons Attribution 4.0 International License from Iaccarino et al. [36].

Right-sided IE, most commonly involving the tricuspid valve, less frequently necessitates surgery. Indications include persistent sepsis despite appropriate antimicrobial therapy, large vegetations exceeding 20 mm, or recurrent pulmonary emboli. In such situations, valve repair is preferred over replacement to reduce the risk of complications associated with prosthetic material in the low-pressure right-sided circulation [39,40]. Tricuspid valve repair (TVr) techniques reduce reinfection in right-sided IE (Figure 4) [36].

Patch repairs for abscess cavities in aortic root infections are generally avoided because of the higher risk of recurrence, paravalvular leaks, and pseudoaneurysm formation. Instead, cavities are excluded from the circulation and left to drain into the pericardial space. When periannular infection extends to the intervalvular fibrosa or central fibrous body, complex reconstructions become necessary, often as the only means of survival. These technically demanding procedures require highly experienced surgeons, which may limit their availability in some centers [2,3,33]. In extremely rare and carefully selected situations where no surgical alternative exists, heart transplantation has been performed as a last resort [41].

Valve Type Selection

Prosthesis selection should be individualized based on patient characteristics, infection profile, and expected compliance. Bioprosthetic valves are often favored in older adults, those with contraindications to anticoagulation, or individuals unlikely to maintain long-term follow-up. These valves eliminate the need for lifelong anticoagulation but are prone to structural degeneration over time [35,42]. Mechanical valves offer superior durability and are typically selected for younger patients who can reliably manage anticoagulation therapy [2].

Special considerations apply in patients with an elevated risk of reinfection, such as those with a history of intravenous drug use, immunosuppression, or prior endocarditis. In such cases, the choice of valve must balance the risk of early structural failure with the practical limitations imposed by patient behavior or comorbidities [2,32,33]. Valve repair is preferred for localized infections due to preservation of native tissue, though it carries a higher reoperation risk compared to mechanical or bioprosthetic replacements [33].

A recent meta-analysis by Flynn et al. [35] compared outcomes of mechanical (n = 2,336) and bioprosthetic (n = 2,057) valve replacement in 4,393 patients with IE, finding no significant difference in overall survival (HR 0.94, 95% CI 0.73-1.21, P = 0.62), reinfection (HR 0.95, 95% CI 0.48-1.89, P = 0.89), or reoperation rates (HR 0.82, 95% CI 0.34-1.98, P = 0.66) [35]. Patients receiving mechanical valves were younger (52.1 vs. 59.2 years, P < 0.001), potentially confounding unadjusted comparisons.

Homografts and autografts offer advantages in complex cases with high reinfection risk, as evidenced by low reinfection rates (0.2-5.5%), but are limited by structural valve degeneration (SVD) and availability [33]. There is no universally superior valve type in IE; instead, individualized decision-making by the surgical team is paramount [2]. Table 3 compares these strategies, highlighting indications, advantages, disadvantages, outcomes, and guideline recommendations.

**Table 3:** Comparison of Surgical Strategies for Infective Endocarditis: Repair and Replacement Options Classes of Recommendation — I: strong recommendation; IIa: moderate recommendation; IIb: weak recommendation. Levels of Evidence — A: high-quality evidence from multiple randomized trials or meta-analyses; B: moderate-quality evidence from a single randomized trial or large non-randomized studies; C: consensus of experts or small/retrospective studies.

Surgical Strategy	Indications/IE Type	Advantages	Disadvantages	Outcomes (Survival, Reinfection, Reoperation)	Guidelines (Class/Level)
Valve Repair (e.g., Vegetectomy, Patch Reconstruction, Ozaki Procedure, Autologous Pericardium) [10,33,43]	Localized infection limited to valve cusps/leaflets; uncomplicated native valve endocarditis (NVE) (e.g., streptococci-treated); mitral as primary site; intact annulus. Preferred for tricuspid/mitral when feasible.	Preserves native tissue; avoids anticoagulation; lower prosthetic complications; feasible in expert centers.	High reoperation rate; limited to experienced surgeons; not suitable for extensive destruction.	Survival: Comparable to replacement in select cases; Reinfection: Low if complete debridement; Reoperation: High (up to 20-30% at 10 years).	Class I, Level B (repair whenever possible for localized NVE/PVE).
Mechanical Replacement [10,33,43-45]	Localized NVE/prosthetic valve endocarditis (PVE) with preserved annulus; younger patients (<50-69 years) compliant with anticoagulation; abscess with mitro-aortic involvement.	Superior durability; low structural valve degeneration (SVD); suitable for reconstruction with patches.	Requires lifelong anticoagulation (bleeding risk 13% at 15 years); contraindicated in stroke/hemorrhage.	Survival: 62.1% at 15 years; Reinfection: 2.1-2.3% at 5 years; Reoperation: Low (6.9% at 15 years).	Class IIa, Level B (for preserved annulus after debridement; avoid in intracranial bleeding, Class IIa, Level C).
Bioprosthetic Replacement (Stented Xenograft, e.g., Carpentier-Edwards Perimount) [10,33,43,46,47]	Localized NVE/PVE; older patients (>60-69 years); anticoagulation contraindications; wish to avoid warfarin.	No anticoagulation needed; easier implantation; suitable for future valve-in-valve transcatheter aortic valve replacement (TAVR).	Higher SVD (starts at eight to 10 years; 45-48.5% freedom at 20 years in young); early failures in some models.	Survival: 60.6% at 15 years; Reinfection: 1.4-2.3% at 20 years; Reoperation: Higher (12.1% at 15 years).	Class IIa, Level B (based on age/life expectancy/comorbidities).
Bioprosthetic Replacement (Stentless Xenograft, e.g., Sorin Freedom Solo) [10,33,48]	Similar to stented; NVE with hemodynamic needs; small roots.	Better hemodynamics (lower gradients); potential lower SVD in some comparisons.	Inferior durability vs. stented (e.g., 5.2% SVD vs. 0% at 6 years); limited data in IE.	Survival: Inferior in NVE (impact of redo); Reinfection: Low (comparable); Reoperation: Higher (9.1% vs. 1.3% at 6 years).	Class IIa, Level B (acceptable alternative; limited IE-specific evidence).
Homograft (Cryopreserved Allograft) [10,33,43,49-52]	Complex/extended IE: Periannular abscess, root destruction, aorto-mitral discontinuity; PVE with aggressive pathogens (e.g., MRSA, fungi); high relapse risk.	Antibiotic-responsive (21-25% medically treatable); antibacterial properties; adapts to irregular tissues; low reinfection.	Early SVD (59.3% freedom at 14 years); reoperation technically demanding (24.8% at 15 years); limited availability; declining use (5.6% in 2011).	Survival: 47-88% at 10 years; Reinfection: Low (0.2-5.5%); Reoperation: 2.2-3.84% for reinfection at 14 years.	Class IIa, Level B (preferred for invasive/destructive AVE; reasonable for root reconstruction).
Autograft (Ross Procedure) [10,33,43,53-55]	Young/middle-aged adults (life expectancy >15 years); women planning pregnancy; high relapse risk (e.g., periannular abscess); contraindications to anticoagulation/prosthetics.	Excellent hemodynamics; growth potential in pediatrics; low valve complications; avoids prosthetic material.	Technical complexity; risk of dual-valve failure; higher operative mortality (three-fold vs. conventional); limited in IE (<200 cases).	Survival: 86.1% at 20 years; Reinfection: Very low (0.4%); Reoperation: Low for reinfection.	Class IIa, Level B (in select young patients at experienced centers).
Other (e.g., Prosthetic Valved Conduits/Bioroots, Sutureless Valves) [10,33,43,44,56,57]	Extensive destruction requiring root replacement; high-risk patients needing short operative times; alternatives when homografts unavailable. Sutureless for redo/high-risk IE.	Customizable for complex anatomy; reduced cross-clamp time (sutureless); acceptable in preserved annulus.	Higher reinfection vs. homografts; limited IE data; not for TAVR IE (contraindicated).	Survival: Comparable to homografts in non-complex; Reinfection: Higher; Reoperation: Variable. Surgery improves survival in post-TAVR IE.	Class IIa, Level B (acceptable alternatives for root reconstruction; sutureless limited evidence).

The Commando Procedure

This is an intricate form of cardiac surgery that entails the concurrent replacement or repair of both the aortic and mitral valves, along with reconstruction of the intervalvular fibrous body, also referred to as the AMC. It is primarily performed in cases of severe IE affecting the AMC. The operation involves complete removal of the diseased valves and any infected or necrotic tissue, followed by AMC reconstruction using materials such as bovine pericardium, Dacron, or autologous pericardium to re-establish structural stability. Mechanical or bioprosthetic valves are then placed in the aortic and mitral positions, with further reconstruction of the aortic root or left atrial roof carried out if necessary [58].

Since its introduction in 1979 by David and colleagues [59], mortality rates for this procedure have remained considerable, with one-year mortality ranging from 20% to 30% and a high incidence of intraoperative complications [60]. In a series reported by Forteza et al. [61], covering the years 1997-2013, 40 consecutive patients underwent IVFB reconstruction, 26 of whom had IE. For those with endocarditis, freedom from reoperation was 84.6% at five years and 76.9% at 10 years, while survival was 57.7% and 50% at the same time points, respectively. The authors concluded that, despite the substantial risks, the procedure is a viable last-resort option when no other surgical alternatives exist, but it necessitates ongoing monitoring due to the possibility of late dehiscence.

**Figure 5:**
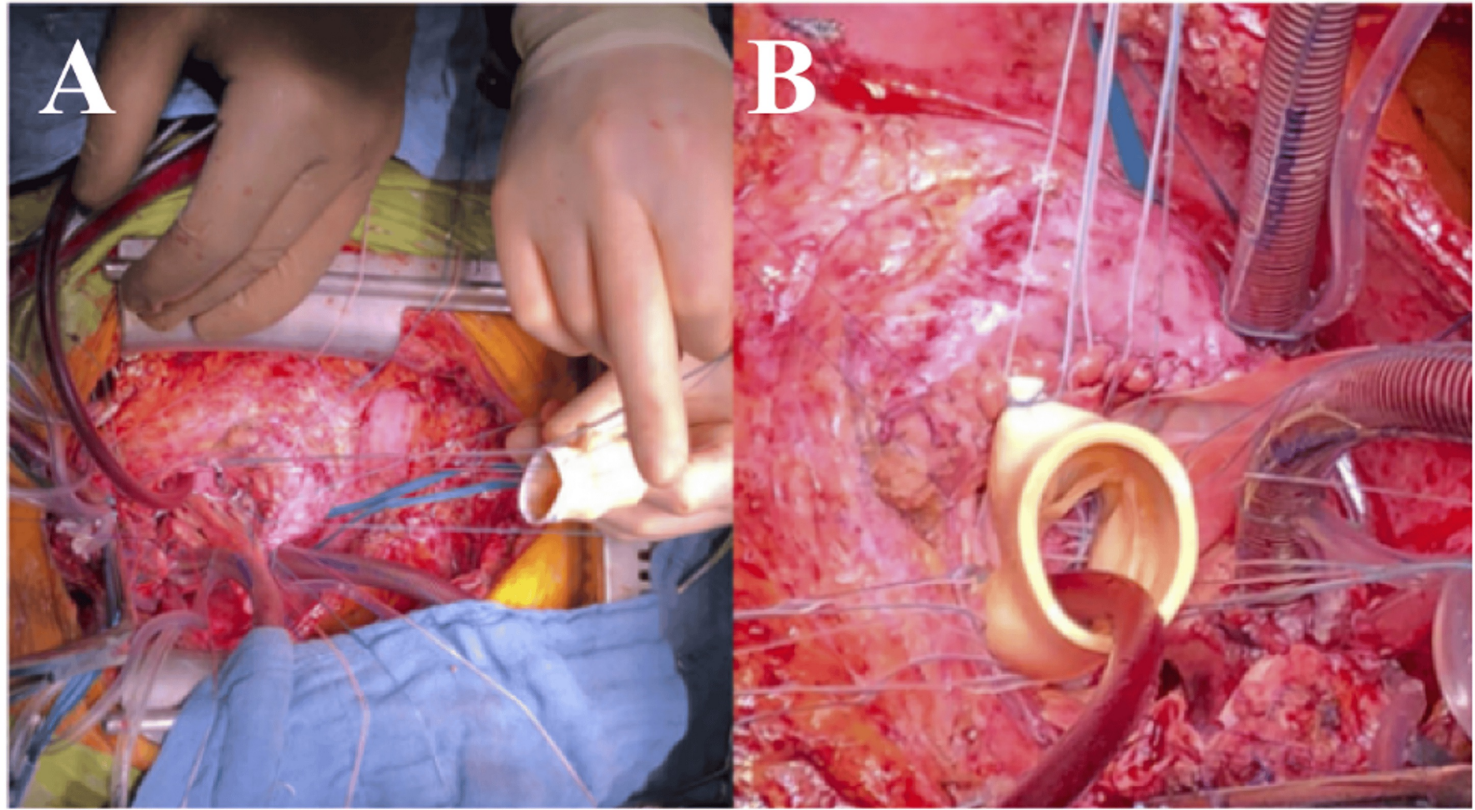
Commando Procedure-Essen Modification A. Conventional implantation technique of a stentless aortic root prosthesis; B. lowering of the stentless aortic root prosthesis into the native aortic root; C. Final postoperative result: implanted aortic root prosthesis and right atrium closed with the anterior aspect of the pericardium patch. Reprinted under the terms of the Creative Commons Attribution 4.0 International License from Zubarevich et al. [63].

In a more recent study, David et al. [62] presented long-term results in 182 consecutive patients treated between 1985 and 2020, reporting an operative mortality of 13.2%, with 10-year and 20-year survival rates of 51.1% and 23.7%, respectively. Nonetheless, reoperation rates remain elevated, approaching 50% within the first five years across the published series. More recently, the Essen team introduced a modified approach, designated the Essen-Commando (Figure 5) [63], that incorporates a Medtronic Freestyle stentless aortic root prosthesis for replacement. They suggest that the prosthesis flexibility contributes to optimal hemostatic control and hemodynamic performance.

Perioperative Challenges

Perioperative management in IE often involves balancing the urgency of surgical intervention with patient-specific risks. A major consideration is the timing of surgery in those with recent neurological events [64]. The current trend in management favors early surgery due to its association with better survival and operative outcomes. However, in patients with recent ischemic or hemorrhagic stroke, determining the optimal timing is complex. Neurological deterioration may occur during or shortly after surgery as a result of the altered physiological conditions of cardiopulmonary bypass and the postoperative state. Therefore, surgical decisions must weigh the risk of exacerbating neurological injury against the consequences of delaying intervention [65,66].

In hemodynamically unstable patients, surgery should not be delayed. Similarly, patients who have experienced a transient ischemic attack generally tolerate prompt surgery with minimal risk. In those with ischemic stroke, evidence supports proceeding urgently unless neurological status is severely impaired, such as in coma or in cases of extensive cerebral damage with a poor prognosis. Collaboration with neurology and neurosurgical teams is essential for individualized risk assessment [67]. Hemorrhagic transformation after surgery occurs in 2% to 7% of patients with prior stroke and can also affect those with clinically silent preoperative cerebral emboli. Such complications are unpredictable and carry high mortality, often requiring urgent neurosurgical intervention for bleeding control or decompression [20,68].

In cases of hemorrhagic stroke, selected patients may still undergo surgery within two weeks without worsening neurological outcomes, provided that case selection is careful and severity is assessed through imaging and validated tools such as the National Institutes of Health Stroke Scale. If surgery is postponed, follow-up neuroimaging should be performed one to two weeks after the event, or earlier if clinical deterioration occurs, to confirm stability before proceeding [67]. In addition to neurological considerations, intraoperative TOE plays a vital role in guiding repair quality and detecting complications in real time [5,69]. Anticoagulation and bleeding risk must be meticulously managed, particularly in patients with recent cerebral events, prosthetic material, or high thromboembolic risk, to optimize both surgical safety and long-term outcomes [5].

Postoperative management and long-term outcomes

Successful surgical intervention in IE marks a critical milestone in recovery but must be followed by vigilant postoperative care and long-term follow-up to prevent recurrence, ensure valve function, and support patient rehabilitation [2,3]. The postoperative phase involves not only medical and surgical monitoring but also psychosocial support and lifestyle modification, all of which contribute to optimizing long-term outcomes [6,70].

Immediate Postoperative Care

Postoperative care in IE is often challenging due to the systemic effects of the infection, preoperative multiorgan involvement, and the complexity of surgical interventions. Postoperative antibiotics should continue for six weeks, adjusted by intraoperative cultures, with prophylaxis for high-risk procedures in PVE patients [6]. Follow-up includes TTE at six weeks, focusing on valve function and recurrence [71].

Despite modern advances, in-hospital mortality after surgical intervention for IE remains significant - typically between 10% and 20% - and is highest in older patients, particularly those over 75 years of age. This elevated risk is usually attributable to underlying comorbidities and the systemic complications of endocarditis. Strategies to reduce surgical mortality in this population remain an important focus for future research [71,72].

Serious postoperative complications are common and include coagulopathy requiring transfusion of blood products and clotting factors, re-exploration for bleeding or tamponade, acute kidney injury requiring hemodialysis, neurological events such as stroke or hemorrhagic transformation of pre-existing lesions, low cardiac output syndrome, and respiratory failure that may necessitate prolonged ventilation or tracheostomy. Extended hospital stays and the need for permanent pacemaker implantation are also frequently encountered [71,72].

When mortality occurs in the postoperative period, it is often the result of multiple interacting factors rather than a single cause. In such cases, post-mortem examination can be invaluable in determining the exact cause of death, improving understanding of the disease process, providing educational opportunities, and contributing to institutional quality control and surgical performance review [73,74].

Recurrence and Reinfection

A key concern in the months and years following surgery for IE is the possibility of disease recurrence. This can present in two distinct forms: relapse and reinfection. Relapse refers to the return of infection by the same microorganism, typically within six months of completing antimicrobial therapy, and is usually due to incomplete eradication during the initial treatment. In contrast, reinfection involves a different microorganism and occurs after a period of apparent cure, often linked to new exposure or risk behaviors [75-77].

A meta-analysis of 652 patients by Flynn et al. [35] found no significant difference in reinfection rates between mechanical and bioprosthetic valves (HR 0.95, 95% CI 0.48-1.89, P = 0.89; I² = 5%). Freedom from reinfection was 97.0% at one year, 91.4% at three years, 88.3% at five years, and 79.0% at 10 years for bioprosthetic valves, compared to 97.1%, 93.8%, 92.3%, and 86.6% for mechanical valves. These findings align with the distinction between relapse (same organism, six months), as reinfection risk is driven by patient factors like ongoing drug use or immunosuppression rather than prosthesis type. Thorough intraoperative debridement and tailored antibiotic therapy remain essential to minimize recurrence risk. Understanding the differences between these two entities is essential for tailoring diagnostic workup, management strategies, and patient counseling. Table 4 below outlines the key distinctions between relapse and reinfection [73,78,79].

**Table 4:** Comparison of Relapse and Reinfection in Postoperative Infective Endocarditis

Feature	Relapse (Recurrence)	Reinfection
Definition	Recurrence of IE due to the same microorganism	New episode of IE due to a different microorganism
Timing	Usually occurs within six months of completing treatment	Occurs after six months, often after a period of clinical cure
Cause	Incomplete eradication of original infection	New infection from a new exposure or source
Common Risk Factors	Inadequate antibiotic therapy, incomplete surgical debridement	Poor dental hygiene, persistent IV drug use, indwelling devices
Microbiologic Features	Identical strain on culture or molecular testing	Different strain or species on repeat cultures
Clinical Implications	Suggests treatment failure or persistence of infection source	Indicates re-exposure; requires renewed source investigation
Treatment Approach	Often requires prolonged antibiotic therapy and possible reoperation	Full evaluation and new tailored treatment regimen
Prognosis	May carry worse prognosis due to resistant organisms or structural damage	Depends on timing, comorbidities, and organism virulence

Follow-Up Strategies

Postoperative follow-up in IE should be structured, proactive, and multidisciplinary. Clinical surveillance includes regular assessment of symptoms, functional capacity, and signs of heart failure or systemic infection [74,80]. Microbiologic monitoring may be necessary in select high-risk patients, while serial echocardiography is vital for assessing valve function and detecting prosthetic complications [81,82].

Outpatient management should ideally be coordinated by a team that includes cardiology, infectious diseases, cardiac surgery, and primary care. Education on the importance of dental and skin hygiene, catheter care, and early reporting of infection symptoms is essential for all patients. Lifestyle modifications, including smoking cessation, diabetes control, and managing vascular risk factors, contribute to both cardiovascular health and infection prevention [83,84].

Rehabilitation and Psychosocial Support

Cardiac rehabilitation should be offered to suitable patients following surgery, as it facilitates recovery of physical function, improves quality of life, and reduces hospital readmission rates. Rehabilitation programs should incorporate nutritional counseling, supervised exercise, and education on medication adherence and lifestyle changes [81,82].

Given the high psychological burden of IE, psychosocial evaluation is also critical. Screening for depression, cognitive decline, and especially substance use disorder is essential, particularly among PWID, who are at increased risk of both reinfection and early mortality. Engagement with addiction medicine, social work, and psychiatric services is often required to support sustained recovery and prevent relapse into high-risk behaviors [74,80].

Long-Term Prognosis

Long-term outcomes after surgical treatment for IE vary widely depending on the underlying pathogen, presence of complications, and comorbid conditions. Survival rates have improved, but late mortality remains significant, often due to heart failure, recurrent infection, or non-cardiac causes such as malignancy or overdose in high-risk populations [73,77].

Prosthetic valve durability is a key consideration in younger patients and those undergoing multiple reoperations. Bioprosthetic valves are prone to structural degeneration over time, whereas mechanical valves offer longevity but require strict anticoagulation adherence. The need for reoperation is higher in patients with persistent risk factors such as ongoing drug use, immunosuppression, or prosthetic-related complications [75,85,86].

Flynn et al. [35] reported no survival difference between mechanical and bioprosthetic valves in 3,884 patients (HR 0.94, 95% CI 0.73-1.21, P = 0.62), with survival rates of 71.8% (bioprosthetic) vs. 73.2% (mechanical) at one year, 57.3% vs. 56.8% at three years, 49.2% vs. 49.3% at five years, and 37.5% vs. 34.0% at 10 years. Reoperation rates were also comparable (HR 0.82, 95% CI 0.34-1.98, P = 0.66), though bioprosthetic valves showed higher reoperation at 10 years (43.1% vs. 24.4%). Late mortality is driven by comorbidities, heart failure, and non-cardiac causes (e.g., overdose in PWID), underscoring the need for structured follow-up and rehabilitation to optimize functional outcomes.

From a functional standpoint, many patients achieve a good quality of life post-surgery, especially when enrolled in structured rehabilitation and follow-up programs. However, those with neurological sequelae, recurrent infections, or significant residual valve dysfunction may experience lasting limitations. Tailored long-term care pathways are essential to meet the diverse needs of this complex patient population [31,87].

Framing future research priorities

Several gaps in evidence, primarily from observational data (most insights at level B or C), highlight the need for RCTs to strengthen the evidence base. Key priorities include the following: (1) RCTs to assess antibiotic prophylaxis efficacy following recent practice shifts, given mixed incidence trends; (2) prospective studies comparing oral versus intravenous antibiotics in stable left-sided IE, including long-term outcomes; (3) trials to evaluate surgical timing after ischemic or hemorrhagic strokes, balancing embolic versus bleeding risks; (4) additional data on IE incidence, characteristics, and management in transcatheter valve therapies (TAVI/TPVI) and left ventricular assist devices; (5) validation of 18F-FDG-PET/CT in native valve IE and routine screening for embolic events; (6) research on gender disparities in IE outcomes, addressing underrepresentation of women in studies; (7) studies in special populations (e.g., elderly, pregnant, immunocompromised, low- and middle-income countries) to clarify tailored prophylaxis and therapy; and (8) long-term follow-up registries to assess relapse/reinfection rates and prosthesis durability. These priorities aim to refine diagnostic approaches, optimize multidisciplinary care, and reduce IE morbidity and mortality through evidence-based interventions [9].

Limitations

This review synthesizes current evidence and expert consensus on the surgical management of IE, but certain limitations must be acknowledged. First, much of the available literature, particularly regarding timing of surgery, valve choice, and management in complex scenarios such as recent neurological injury, derives from observational studies rather than RCTs. As such, conclusions may be influenced by selection bias, heterogeneous patient populations, and institutional variations in practice.

Second, the surgical techniques and outcomes discussed are often based on experiences from high-volume, specialized centers, which may limit generalizability to lower-volume institutions or resource-limited settings. Furthermore, some recommendations reflect expert opinion in areas where robust comparative data are lacking, particularly in pediatric populations, PVE, and right-sided disease.

Finally, while this article aims to provide a comprehensive overview, it is not an exhaustive systematic review, and emerging evidence published after the time of writing may refine or alter some of the perspectives presented. Continued high-quality research and multicenter collaboration are essential to address these gaps and strengthen the evidence base for surgical decision-making in IE.

## Conclusions

Surgical management remains a cornerstone in the treatment of IE, providing life-saving intervention for patients with heart failure, uncontrolled infection, or high embolic risk. Optimal outcomes depend on the timely identification of surgical indications, careful perioperative evaluation, and individualized selection of operative techniques such as repair, replacement, or complex reconstructions. Advances in imaging and perioperative strategies have improved risk stratification and surgical planning, yet postoperative mortality, recurrence, and reinfection continue to present significant challenges. A multidisciplinary approach that brings together cardiology, cardiac surgery, infectious diseases, neurology, and rehabilitation is essential to meet both the acute and long-term needs of this high-risk population. Continued research through prospective studies and multicenter collaboration is necessary to refine surgical timing, prosthesis selection, and long-term follow-up strategies. Ultimately, comprehensive patient-centered care that extends beyond the operating room is critical to improving survival, quality of life, and functional outcomes in patients with IE.

## References

[1] Habib G, Badano L, Tribouilloy C (2010). Recommendations for the practice of echocardiography in infective endocarditis. Eur J Echocardiogr.

[2] Pettersson GB, Hussain ST (2019). Current AATS guidelines on surgical treatment of infective endocarditis. Ann Cardiothorac Surg.

[3] Pettersson GB, Coselli JS, Pettersson GB (2017). 2016 The American Association for Thoracic Surgery (AATS) consensus guidelines: surgical treatment of infective endocarditis: executive summary. J Thorac Cardiovasc Surg.

[4] Cahill TJ, Prendergast BD (2016). Infective endocarditis. Lancet.

[5] Jassal DS, Neilan TG, Pradhan AD, Lynch KE, Vlahakes G, Agnihotri AK, Picard MH (2006). Surgical management of infective endocarditis: early predictors of short-term morbidity and mortality. Ann Thorac Surg.

[6] Arjomandi Rad A, Zubarevich A, Osswald A (2024). The surgical treatment of infective endocarditis: a comprehensive review. Diagnostics (Basel).

[7] Rao VP, Wu J, Gillott R, Baig MW, Kaul P, Sandoe JA (2019). Impact of the duration of antibiotic therapy on relapse and survival following surgery for active infective endocarditis. Eur J Cardiothorac Surg.

[8] Ramos-Martínez A, Calderón-Parra J, Miró JM (2019). Effect of the type of surgical indication on mortality in patients with infective endocarditis who are rejected for surgical intervention. Int J Cardiol.

[9] Habib G, Lancellotti P, Antunes MJ (2015). 2015 ESC Guidelines for the management of infective endocarditis: the Task Force for the Management of Infective Endocarditis of the European Society of Cardiology (ESC). Endorsed by: European Association for Cardio-Thoracic Surgery (EACTS), the European Association of Nuclear Medicine (EANM). Eur Heart J.

[10] Vahanian A, Beyersdorf F, Praz F (2022). 2021 ESC/EACTS Guidelines for the management of valvular heart disease. Eur Heart J.

[11] Iung B, Doco-Lecompte T, Chocron S (2016). Cardiac surgery during the acute phase of infective endocarditis: discrepancies between European Society of Cardiology guidelines and practices. Eur Heart J.

[12] Mir T, Uddin M, Qureshi WT, Regmi N, Tleyjeh IM, Saydain G (2022). Predictors of complications secondary to infective endocarditis and their associated outcomes: a large cohort study from the National Emergency Database (2016-2018). Infect Dis Ther.

[13] Pericàs JM, Hernández-Meneses M, Muñoz P (2021). Characteristics and outcome of acute heart failure in infective endocarditis: focus on cardiogenic shock. Clin Infect Dis.

[14] Østergaard L, Dahl A, Bruun NE (2020). Valve regurgitation in patients surviving endocarditis and the subsequent risk of heart failure. Heart.

[15] Bonou MS, Papadimitraki ED, Kapelios CJ, Barbetseas J, Viniou NA (2020). Unusual complications of infective endocarditis. Am J Case Rep.

[16] Park LP, Chu VH, Peterson G (2016). Validated risk score for predicting 6-month mortality in infective endocarditis. J Am Heart Assoc.

[17] Habib G, Erba PA, Iung B (2019). Clinical presentation, aetiology and outcome of infective endocarditis. Results of the ESC-EORP EURO-ENDO (European infective endocarditis) registry: a prospective cohort study. Eur Heart J.

[18] Calderón Parra J, De Castro-Campos D, Muñoz García P (2021). Non-HACEK gram negative bacilli endocarditis: analysis of a national prospective cohort. Eur J Intern Med.

[19] Fernández-Hidalgo N, Ribera A, Larrosa MN (2018). Impact of Staphylococcus aureus phenotype and genotype on the clinical characteristics and outcome of infective endocarditis. A multicentre, longitudinal, prospective, observational study. Clin Microbiol Infect.

[20] Diab M, Guenther A, Sponholz C (2016). Pre-operative stroke and neurological disability do not independently affect short- and long-term mortality in infective endocarditis patients. Clin Res Cardiol.

[21] Selton-Suty C, Delahaye F, Tattevin P (2016). Symptomatic and asymptomatic neurological complications of infective endocarditis: impact on surgical management and prognosis. PLoS One.

[22] Ferrera C, Vilacosta I, Fernández C (2018). Early surgery for acute-onset infective endocarditis. Eur J Cardiothorac Surg.

[23] Mohananey D, Mohadjer A, Pettersson G (2018). Association of vegetation size with embolic risk in patients with infective endocarditis: a systematic review and meta-analysis. JAMA Intern Med.

[24] Kim YK, Choi CG, Jung J (2018). Effect of cerebral embolus size on the timing of cardiac surgery for infective endocarditis in patients with neurological complications. Eur J Clin Microbiol Infect Dis.

[25] Dashkevich A, Bratkov G, Li Y (2021). Impact of operative timing in infective endocarditis with cerebral embolism-the risk of intermediate deterioration. J Clin Med.

[26] Diab M, Sponholz C, von Loeffelholz C (2017). Impact of perioperative liver dysfunction on in-hospital mortality and long-term survival in infective endocarditis patients. Infection.

[27] Armiñanzas C, Fariñas-Alvarez C, Zarauza J (2019). Role of age and comorbidities in mortality of patients with infective endocarditis. Eur J Intern Med.

[28] Knol WG, Wahadat AR, Roos-Hesselink JW, Van Mieghem NM, Tanis W, Bogers AJ, Budde RP (2021). Screening for coronary artery disease in early surgical treatment of acute aortic valve infective endocarditis. Interact Cardiovasc Thorac Surg.

[29] Spanneut TA, Paquet P, Bauters C (2022). Utility and safety of coronary angiography in patients with acute infective endocarditis who required surgery. J Thorac Cardiovasc Surg.

[30] Defauw RJ, Tomšič A, van Brakel TJ, Marsan NA, Klautz RJ, Palmen M (2020). A structured approach to native mitral valve infective endocarditis: is repair better than replacement?. Eur J Cardiothorac Surg.

[31] Toyoda N, Itagaki S, Tannous H, Egorova NN, Chikwe J (2018). Bioprosthetic versus mechanical valve replacement for infective endocarditis: focus on recurrence rates. Ann Thorac Surg.

[32] Perrotta S, Zubrytska Y (2016). Valve selection in aortic valve endocarditis. Kardiochir Torakochirurgia Pol.

[33] Nappi F, Singh SS, Spadaccio C, Acar C (2020). Revisiting the guidelines and choice the ideal substitute for aortic valve endocarditis. Ann Transl Med.

[34] Toyoda N, Itagaki S, Egorova NN (2017). Real-world outcomes of surgery for native mitral valve endocarditis. J Thorac Cardiovasc Surg.

[35] Flynn CD, Curran NP, Chan S (2019). Systematic review and meta-analysis of surgical outcomes comparing mechanical valve replacement and bioprosthetic valve replacement in infective endocarditis. Ann Cardiothorac Surg.

[36] Iaccarino A, Barbone A, Basciu A (2023). Surgical challenges in infective endocarditis: state of the art. J Clin Med.

[37] Weymann A, Merzah AS, Arjomandi Rad A (2024). Surgical therapy of infective prosthesis endocarditis following TAVI: a single center's experience. Diagnostics (Basel).

[38] Witten JC, Houghtaling PL, Shrestha NK, Gordon SM, Jaber W, Blackstone EH, Pettersson GB (2023). Aortic allograft infection risk. J Thorac Cardiovasc Surg.

[39] Hussain ST, Witten J, Shrestha NK, Blackstone EH, Pettersson GB (2017). Tricuspid valve endocarditis. Ann Cardiothorac Surg.

[40] Yanagawa B, Elbatarny M, Verma S, Hill S, Mazine A, Puskas JD, Friedrich JO (2018). Surgical management of tricuspid valve infective endocarditis: a systematic review and meta-analysis. Ann Thorac Surg.

[41] Murphy KM, Vikram HR (2019). Heart transplantation for infective endocarditis: viable option for a limited few?. Transpl Infect Dis.

[42] Newton S, Hunter S (2010). What type of valve replacement should be used in patients with endocarditis?. Interact Cardiovasc Thorac Surg.

[43] Otto CM, Nishimura RA, Bonow RO (2021). 2020 ACC/AHA guideline for the management of patients with valvular heart disease: executive summary: a report of the American College of Cardiology/American Heart Association Joint Committee on Clinical Practice Guidelines. Circulation.

[44] David TE, Gavra G, Feindel CM, Regesta T, Armstrong S, Maganti MD (2007). Surgical treatment of active infective endocarditis: a continued challenge. J Thorac Cardiovasc Surg.

[45] Kim JB, Ejiofor JI, Yammine M (2016). Are homografts superior to conventional prosthetic valves in the setting of infective endocarditis involving the aortic valve?. J Thorac Cardiovasc Surg.

[46] Johnston DR, Soltesz EG, Vakil N (2015). Long-term durability of bioprosthetic aortic valves: implications from 12,569 implants. Ann Thorac Surg.

[47] Bourguignon T, Bouquiaux-Stablo AL, Candolfi P (2015). Very long-term outcomes of the Carpentier-Edwards Perimount valve in aortic position. Ann Thorac Surg.

[48] Schaefer A, Dickow J, Schoen G (2018). Stentless vs. stented bioprosthesis for aortic valve replacement: a case matched comparison of long-term follow-up and subgroup analysis of patients with native valve endocarditis. PLoS One.

[49] Steffen V, Marsch G, Burgwitz K, Kuehn C, Teebken OE (2016). Resistance to infection of long-term cryopreserved human aortic valve allografts. J Thorac Cardiovasc Surg.

[50] Musci M, Weng Y, Hübler M (2010). Homograft aortic root replacement in native or prosthetic active infective endocarditis: twenty-year single-center experience. J Thorac Cardiovasc Surg.

[51] Arabkhani B, Bekkers JA, Andrinopoulou ER, Roos-Hesselink JW, Takkenberg JJ, Bogers AJ (2016). Allografts in aortic position: insights from a 27-year, single-center prospective study. J Thorac Cardiovasc Surg.

[52] Fukushima S, Tesar PJ, Pearse B, Jalali H, Sparks L, Fraser JF, Pohlner PG (2014). Long-term clinical outcomes after aortic valve replacement using cryopreserved aortic allograft. J Thorac Cardiovasc Surg.

[53] Ratschiller T, Sames-Dolzer E, Paulus P, Schimetta W, Müller H, Zierer AF, Mair R (2017). Long-term evaluation of the Ross procedure in acute infective endocarditis. Semin Thorac Cardiovasc Surg.

[54] El-Hamamsy I, Eryigit Z, Stevens LM (2010). Long-term outcomes after autograft versus homograft aortic root replacement in adults with aortic valve disease: a randomised controlled trial. Lancet.

[55] Stelter J, Goot BH (2025). Infective endocarditis of the autograft and the neo-aortic root following the Ross operation. CASE (Phila).

[56] Fujimoto R, Hirao S, Komiya T (2025). Infective endocarditis with perivalvular abscess following sutureless valve implantation, successfully treated with aortic root reconstruction and biological Bentall procedure. J Cardiothorac Surg.

[57] Magouliotis DE, Sicouri S, Baudo M, Cabrucci F, Yamashita Y, Ramlawi B (2025). Surgical vs. medical management of infective endocarditis following TAVR: a systematic review and meta-analysis. J Cardiovasc Dev Dis.

[58] Chen L, Mahboubi R, Kakavand M, Erten O, Blackstone EH, Johnston DR (2022). Improvements in outcomes and expanding indications for the Commando procedure. Comment on Giambuzzi et al. Surgical aortic mitral curtain replacement: systematic review and metanalysis of early and long-term results. J Clin Med.

[59] David TE, Kuo J, Armstrong S, Carpentier AF, Magotra RA (1997). Aortic and mitral valve replacement with reconstruction of the intervalvular fibrous body. J Thorac Cardiovasc Surg.

[60] Davierwala PM, Binner C, Subramanian S (2014). Double valve replacement and reconstruction of the intervalvular fibrous body in patients with active infective endocarditis. Eur J Cardiothorac Surg.

[61] Forteza A, Centeno J, Ospina V (2015). Outcomes in aortic and mitral valve replacement with intervalvular fibrous body reconstruction. Ann Thorac Surg.

[62] David TE, Lafreniere-Roula M, David CM, Issa H (2022). Outcomes of combined aortic and mitral valve replacement with reconstruction of the fibrous skeleton of the heart. J Thorac Cardiovasc Surg.

[63] Zubarevich A, Zhigalov K, Osswald A (2021). Essen-Commando: how we do it. J Card Surg.

[64] Zhang LQ, Cho SM, Rice CJ (2020). Valve surgery for infective endocarditis complicated by stroke: surgical timing and perioperative neurological complications. Eur J Neurol.

[65] Matthews CR, Hartman T, Madison M (2021). Preoperative stroke before cardiac surgery does not increase risk of postoperative stroke. Sci Rep.

[66] Okita Y, Minakata K, Yasuno S (2016). Optimal timing of surgery for active infective endocarditis with cerebral complications: a Japanese multicentre study. Eur J Cardiothorac Surg.

[67] Ragulojan R, Grupke S, Fraser JF (2019). Systematic review of endovascular, surgical, and conservative options for infectious intracranial aneurysms and cardiac considerations. J Stroke Cerebrovasc Dis.

[68] Jiad E, Gill SK, Krutikov M, Turner D, Parkinson MH, Curtis C, Werring DJ (2017). When the heart rules the head: ischaemic stroke and intracerebral haemorrhage complicating infective endocarditis. Pract Neurol.

[69] Koo HJ, Yang DH, Kang JW (2018). Demonstration of infective endocarditis by cardiac CT and transoesophageal echocardiography: comparison with intra-operative findings. Eur Heart J Cardiovasc Imaging.

[70] Wang A, Gaca JG, Chu VH (2018). Management considerations in infective endocarditis: a review. JAMA.

[71] Wang A, Chu VH, Athan E (2019). Association between the timing of surgery for complicated, left-sided infective endocarditis and survival. Am Heart J.

[72] Hill TE, Kiehl EL, Shrestha NK (2021). Predictors of permanent pacemaker requirement after cardiac surgery for infective endocarditis. Eur Heart J Acute Cardiovasc Care.

[73] Scheggi V, Merilli I, Marcucci R (2021). Predictors of mortality and adverse events in patients with infective endocarditis: a retrospective real world study in a surgical centre. BMC Cardiovasc Disord.

[74] Kimmel SD, Walley AY, Li Y (2020). Association of treatment with medications for opioid use disorder with mortality after hospitalization for injection drug use-associated infective endocarditis. JAMA Netw Open.

[75] Havers-Borgersen E, Butt JH, Østergaard L (2020). Recurrent infective endocarditis versus first-time infective endocarditis after heart valve surgery. Clin Res Cardiol.

[76] Citro R, Chan KL, Miglioranza MH (2022). Clinical profile and outcome of recurrent infective endocarditis. Heart.

[77] Yoshioka D, Toda K, Yokoyama JY (2018). Diabetes mellitus adversely affects mortality and recurrence after valve surgery for infective endocarditis. J Thorac Cardiovasc Surg.

[78] Agrawal A, Virk HU, Riaz I (2020). Predictors of 30-day re-admissions in patients with infective endocarditis: a national population based cohort study. Rev Cardiovasc Med.

[79] Freitas-Ferraz AB, Tirado-Conte G, Vilacosta I (2020). Contemporary epidemiology and outcomes in recurrent infective endocarditis. Heart.

[80] Price CN, Solomon DA, Johnson JA, Montgomery MW, Martin B, Suzuki J (2020). Feasibility and safety of outpatient parenteral antimicrobial therapy in conjunction with addiction treatment for people who inject drugs. J Infect Dis.

[81] Rasmussen TB, Zwisler AD, Risom SS (2022). Comprehensive cardiac rehabilitation for patients following infective endocarditis: results of the randomized CopenHeartIE trial. Eur J Cardiovasc Nurs.

[82] Rasmussen TB, Zwisler AD, Thygesen LC, Bundgaard H, Moons P, Berg SK (2017). High readmission rates and mental distress after infective endocarditis — results from the national population-based CopenHeart IE survey. Int J Cardiol.

[83] Rasmussen TB, Zwisler AD, Moons P, Berg SK (2015). Insufficient living: experiences of recovery after infective endocarditis. J Cardiovasc Nurs.

[84] Shah AS, McAllister DA, Gallacher P (2020). Incidence, microbiology, and outcomes in patients hospitalized with infective endocarditis. Circulation.

[85] Straw S, Baig MW, Gillott R, Wu J, Witte KK, O'regan DJ, Sandoe JA (2020). Long-term outcomes are poor in intravenous drug users following infective endocarditis, even after surgery. Clin Infect Dis.

[86] Abegaz TM, Bhagavathula AS, Gebreyohannes EA, Mekonnen AB, Abebe TB (2017). Short- and long-term outcomes in infective endocarditis patients: a systematic review and meta-analysis. BMC Cardiovasc Disord.

[87] Østergaard L, Dahl A, Fosbøl E (2019). Residual vegetation after treatment for left-sided infective endocarditis and subsequent risk of stroke and recurrence of endocarditis. Int J Cardiol.

